# Optimization of Cr (VI) removal from aqueous solution with activated carbon derived from *Eichhornia crassipes* under response surface methodology

**DOI:** 10.1186/s13065-023-00913-6

**Published:** 2023-02-13

**Authors:** Jemal Fito, Solomon Tibebu, Thabo T. I. Nkambule

**Affiliations:** 1 Institute for Nanotechnology and Water Sustainability (iNanoWS), College of Science, Engineering and Technology, Florida Science Campus, University of South Africa, Johannesburg, 1710 South Africa; 2 Department of Environmental Engineering, College of Biological and Chemical Engineering, Sustainable Energy Center of Excellence, Bioprocess and Biotechnology Center of Excellence, Addis Ababa Science and Technology University, 16417 Addis Ababa, Ethiopia

**Keywords:** Adsorbent, Environment, Effluent, Removal, Pollutant, Treatment performance

## Abstract

Tannery industries’ effluent contains a high concentration of Cr (VI) which has the potential to affect the environment and public health. Therefore, this study aimed to investigate the optimization of Cr (VI) adsorption by activated carbon (AC) derived from Eichhornia crassipes from an aqueous solution. The adsorbent was activated with dilute sulfuric acid followed by thermal activation. AC was characterized using proximate analysis, SEM, FTIR, X-ray diffraction, and the BET method. The Cr (VI) removal optimization process was performed using a central composite design under the response surface methodology. The proximate analysis showed that the moisture content, volatile matter, ash content, and fixed carbon of the activated carbon were 5.6%, 18.2%, 14.4%, and 61.8% respectively. The surface areas of the Eichhornia crassipes before activation, after activation, and after adsorption were 60.6 g/m^2^, 794.2 g/m^2^, and 412.6 g/m^2^ respectively. A highly porous structure with heterogeneous and irregular shapes was observed in the SEM micrograph. In the FTIR analysis, different peaks are indicated with various functional groups. The intensity of XRD peaks decreased as 2 theta values increased, which indicates the presence of an amorphous carbon arrangement. The point of zero charge (pH_pzc_) of the activated carbon was found to be 5.20. A maximum Cr (VI) removal of 98.4% was achieved at pH 5, contact time 90 min, adsorbent dose 2 g, and initial Cr (VI) concentration of 2.25 mg/L. Statistically significant interactions (P < 0.05) were observed between the initial Cr (VI) concentration and adsorbent dose as well as the initial Cr (VI) concentration and contact time. Langmuir adsorption isotherm fitted the experimental data best, with an R^2^ value of 0.99. The separation constant (RL) indicates that the adsorption process is favorable. The kinetic experimental data were best fitted with the pseudo-second-order model with an R^2^ value of 0.99 whereas the adsorption rate is controlled by intraparticle and extragranular diffusion processes. Generally, the AC has the potential to be a strong adsorbent candidate for wastewater treatment at the industrial level.

## Introduction

Clean water, public health, and sanitation services are basic human rights. Sustainable development depends on adequate and equitable water and sanitation levels [1]. The United Nations suggested 17 goals of development which were framed for global nations toward acquiring sustainability. Goal 6 is the water availability, quality, and affordability of basic sanitation for all mankind [2]. However, water is being polluted due to various point and non-point sources [3]. Industrial wastes are the major point source of water pollution [4]. Numerous contaminants are being discharged into the environment via industrial effluents, threatening the survival of living organisms worldwide. Chromium is the most known inorganic pollutant in industrial discharges [5, 6]. The discharge of this pollutant is a deteriorating 3% of the global freshwater, contributing to water scarcity in developing countries [7]. This challenge is exacerbated by industrialization, urbanization, and rapid population growth which are contributing to the rapidly increasing water demand [8]. In line with the water demand, about 3–5 billion people are expected to be affected by severe water scarcity by 2050 [9].

Among the various heavy metals, chromium is a common pollutant in surface and groundwater [10]. The two oxidation states of chromium in the environment are trivalent chromium (Cr (III)) and hexavalent chromium (Cr(VI)) [11]. Cr (VI) is an exceedingly poisonous heavy metal that is about 500 times more toxic than Cr (III), which is substantially less toxic to humans [12]. Chromium is used in the tannery, cement, textile, electroplating, and mining industries, nuclear power plants, wood preservation, and chromate preparation sectors (VI) [13]. Most tannery industries use the chromium-tanning method because of its speed, low cost, and increased leather stability. As a result, tanning is one of the world's top chromium polluters [14]. Chromium is used in some of the unit operations and processes in tanning such as soaking, washing, liming, de-liming, pickling, chrome tanning, and neutralization [15]. Each of these activities and processes produces a significant volume of effluent which contains high levels of Cr(VI)) [16]. Cr(VI) has hazardous and poisonous properties such as persistence, toxicity, biomagnification, and bioaccumulation in the food chain, all of which impact the environment and public health [17]. It particularly affects organs such liver, lungs, and kidneys. It also causes vomiting, pulmonary congestion, and diarrhea. Its mutagenic and teratogenic effects are of great concern [18]. USEPA regulates Cr (VI) concentrations in drinking water and surface water to the level of 0.05 and 0.1 mg/L, respectively [19, 20]. Treatment of wastewater containing Cr (VI) before releasing it into the environment is the most effective form of prevention.

Traditional wastewater treatment technologies, which include preliminary, primary, secondary, and tertiary treatment stages, are unable to remove effectively Cr (VI) from industrial effluent [21]. As a result, studies are being carried out on the removal of micropollutants including Cr (VI) from industrial effluents. Utilizing advanced wastewater treatment technologies is essential to apply for environmental clean-up [22]. The most often used advanced wastewater treatment methods are electrodialysis, electrocoagulation, membrane separation, ion exchange, reverse osmosis, adsorption, advanced oxidation, liquid extraction, and chemical precipitation [23]. These treatment systems, on the other hand, are extremely costly in terms of energy, chemicals, and operation [5]. In some cases, the efficiencies of these methods are low in addition to a substantial mass production of sludge. These technologies are therefore not suitable for developing countries, including Ethiopia [24]. Studies are being carried out to find cost-effective and efficient advanced wastewater treatment technology [7, 25]. Activated carbon has a significant potential for adsorbing several heavy metals, including Cr (VI) from industrial wastewater [26, 27]. Adsorption is an appealing and desirable approach for removing heavy metals from wastewater because of its ease of design, high efficiency, environmental friendliness, recyclability, and low operational cost [28, 29]. In line with this, commercial activated carbon has a high treatment efficiency for pollutant removal but because of its high production cost, this technology is not appropriate technology for developing countries [30]. Thus, researchers are still looking for activated carbon that is low in cost, readily available, efficient, and rich in carbon content. Biomass carbon-based adsorbent has been prepared from low-cost such as bagasse fly ash, orange peel, cassava peel, wheat straw, avocado seed, sawdust, the bark of *Moringa* tincture, cocus status leaves, winemaking waste, apricot stine, almond shells, and pine cones [31–33]. These products have limited treatment performance, preparation time, and mass production. Hence, the search for efficient and effective adsorbents for chromium is still under investigation.

*Eichhornia crassipes* (water hyacinth), which is a free-floating perennial aquatic weed, originated from the Amazon basin and spread fast around the world [34]. Water hyacinth is a well-known plant that has a high growth rate. It lowers water quality and impacts the aquatic ecosystem by lowering dissolved oxygen levels and obstructing light penetration into water bodies which impacts aquatic organisms [35]. Furthermore, it negatively affects fisheries, agricultural operations, hydroelectric power plants, and aquatic transportation [36, 37]. The typically stagnant waters infested with water hyacinths are also breeding grounds for disease-transmitting mosquitoes and snails [38]. In Ethiopia, water hyacinth has spread in several bodies, including Lake Tana [39]. Different methods, including manual control and the use of harvester machines, have been used to control water hyacinth, with variable effects. Manual control is the most frequently used method in Ethiopia [34, 35, 39]. Another issue is how to dispose of the weed in an environmentally responsible manner after it has been removed. If the weed is removed and dumped carelessly on the ground, it will take up much space and raise the cost of solid waste management [35]. The adsorption of Cr(VI) from an aqueous solution by activated carbon that is derived from various materials has been studied extensively [40–42]. Haroon et al. [43] reported a maximum Cr (VI) removal efficiency of 87% using activated carbon derived from wood biomass [43]. A maximum Cr (VI) removal efficiency of 97% was achieved using activated carbon derived from paper sludge [44]. It was also reported a maximum adsorption capacity of 188.5 mg/g using activated carbon derived from *luffa cylindrica* [45]. Moreover, many studies have been undertaken to evaluate the potential of employing water hyacinth (*Eichhornia crassipes*) as an adsorbent for chromium removal from wastewater [46–48]. However, to the best of the authors' knowledge, no research has been done on the optimization of Cr(VI) adsorption by activated carbon derived from *Eichhornia crassipes*. Therefore, this study aimed to investigate the optimization of Cr(VI) adsorption by activated carbon (AC) derived from *Eichhornia crassipes* from an aqueous solution. The optimization process is also validated by studying the effect of individual factors (pH, dose, contact time, and initial Cr (VI) concentration).

## Materials and methods

### Adsorbent preparation

**Fig. 1:**
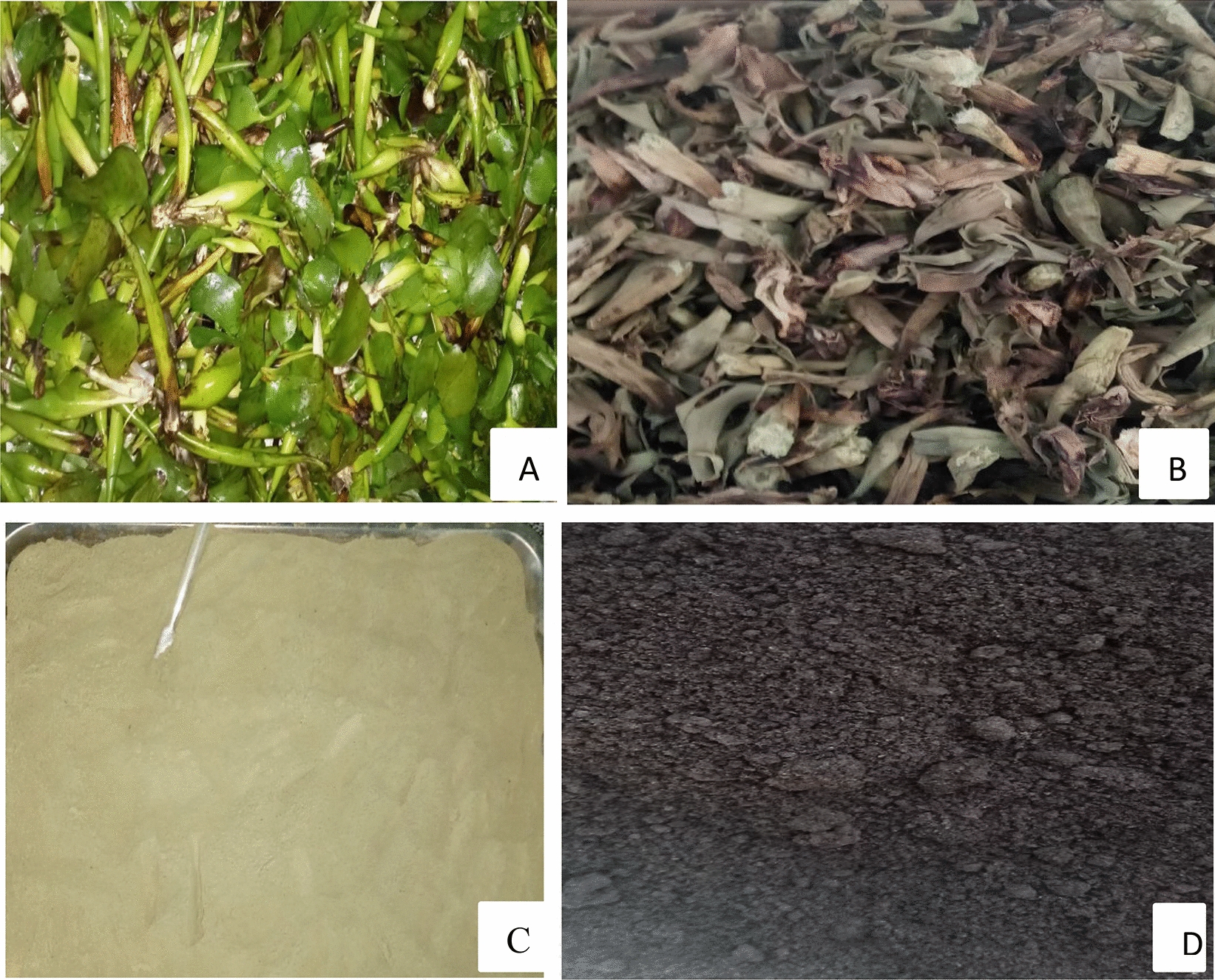
The different stages of AC preparation **A**–**D**. Where ‘**A**’ indicates the raw Erichornia crassipes, ‘**B**’ indicates the dried Erichornia crassipes, ‘**C**’ indicates the crushed Erichornia crassipes, and ‘D’ indicates the final product (AC)

Stems of *Eichhornia crassipes* were collected from Lake Tana, Amhara Regional State, Ethiopia. The voucher plant specimen was deposited in a university herbarium (Addis Ababa University) and the plant specimen was collected by the researcher (Solomon Tibabu) and crossed checked against the herbarium. The plant identification was performed by an expert assigned to the herbarium site. The sample was then cut into small pieces and washed several times with distilled water to remove adhering soil and plant materials. The sample was then dried in an oven at 105 °C for 24 h. The sample was chemically activated by impregnating it with 0.1 M H_2_SO_4_ at a 1:1 acid-to-sample ratio. The activation was conducted at mixing speed, activation time, and activation temperature of 200 rpm, 2 h, and 30 °C respectively. Then, the sample was thermally activated by placing it in a muffle furnace at 500 °C for 2 h with a heating rate of 25 °C/min. The AC was washed with distilled water until the supernatant solution had a pH near 7. The activated carbon was then dried in an oven at $105 ^\circ \mathrm{C}$ for 24 h. Finally, the dried sample was grounded using a high-speed grinder and sieved using a 125 µm sieve. The prepared AC was then placed in an air-tight plastic bag for further use [49, 50]. The AC preparation stages are shown in Fig. 1.

### Characterization of the adsorbent

#### Proximate analysis

The analysis was conducted according to the American Society for Testing and Materials (ASTM) standards as used in many adsorption studies [24, 51, 52]. The moisture content (MC) of the AC was determined according to ASTM (D2867-09) using the oven drying method. About 2 g of AC was placed in a crucible, dried in an oven at $105 ^\circ \mathrm{C}$ for 3 h, and cooled in a desiccator to balance its weight. Then, its weight was measured in an analytical balance (Sartorius BSA224S-CW). The moisture content of the AC was calculated using the following mass balance equation.

1 $$ {\text{MC}}\left( {\text{\% }} \right) = \left( {\frac{{{\text{B}} - {\text{C}}}}{{{\text{B}} - {\text{A}}}}} \right){*}100 $$

where MC is the moisture content of AC in percentage, A is the weight of the crucible (g), B is the weight of the crucible plus the original sample (g) and C is the weight of the crucible plus the oven-dried sample (g).

The volatile matter (VM) of the AC was determined according to ASTM (D5832-98). One gram of AC was placed in a crucible and ignited in a muffle furnace (Nabertherm F 330) at $950 ^\circ \mathrm{C}$ for 7 min. The sample was then cooled in a desiccator and weighted using an electronic balance. The volatile matter of the sample was calculated using the following mass balance equation:

2 $$ {\text{VM}}\left( {\text{\% }} \right) = \left( {\frac{{{\text{D}} - {\text{B}}}}{{{\text{C}} - {\text{B}}}}} \right){*}100 $$

where VM is the volatile matter content of AC in percentage, D is the weight of the crucible and the ignited sample (g), C is the weight of the crucible and the original sample (g), and B is the weight of the crucible (g).

The ash content (AC) of the AC was determined according to ASTM (D2866-94). One gram of AC was placed in a crucible and ignited in a muffle furnace at $500 ^\circ \mathrm{C}$ for 4 h. The sample was then cooled in a desiccator and weighted using an electronic balance. The ash content of the sample was calculated using the following mass balance equation.

3 $$ {\text{AC }}\left( {\text{\% }} \right) = \left( {\frac{{{\text{D}} - {\text{B}}}}{{{\text{C}} - {\text{B}}}}} \right){*}100 $$

where B is the weight of the crucible (g), C is the weight of the crucible plus the original activated carbon sample (g), and D is the weight of the crucible plus the ash sample (g).

The fixed carbon of AC was calculated by using the following equation:

4 $$ {\text{FC}}\left( {\text{\% }} \right) = 100 - \left( {{\text{MC}}\left( {\text{\% }} \right) - {\text{VM}}\left( {\text{\% }} \right) - {\text{AC}}\left( {\text{\% }} \right)} \right) $$

where FC is the fixed carbon content in percentage, MC is moisture content in percentage, VC is the volatile matter content in percent and AC is ash content in percent.

#### Scanning electron microscope (SEM)

SEM was used to determine the morphological characteristics of the AC. The morphological characteristics of the raw and prepared activated carbon were determined at different resolutions. The small amount of AC adsorbent was placed on sample holder and coated under the vacuum compartment of the SEM machine. Then the sample was analyzed under intended condition using SEM machine. Standard operating procedures of SEM (FEI Inspect F50, USA) were used during sample preparation and scanning of the two samples, which were operated at 5.00 kV [53, 54].

#### Fourier transform infrared spectroscopy (FTIR)

FTIR spectrophotometer was used to determine the functional group of the AC. The samples were mixed with KBr at a ratio of 2:200, crushed using mortar, placed on a ZnSe crystal, and pressure was applied [55]. The functional group of AC before and after adsorption was analyzed using FTIR spectrophotometer (Shimadzu IRAffinity-1 s, Japan) in the spectral range of 4000–400 cm^−1^. Finally, the data were analyzed by using Origin Software (Version 9.55) [5, 56, 57].

#### X-ray diffraction (XRD)

The mineral species of the activated carbon were studied using an XRD instrument (Olympus BTXH, Japan) to determine the presence of amorphous and crystalline structures of the AC with a diffraction angle of 2 $\theta$ from 5 to 90º [51]. The X-ray was operated at a current of 15 mA and Cu Kα, 40 kV/40 mA. The scan rate of the X-ray diffraction patterns was 4.2 °C/min [5]. Finally, the data were analyzed using Origin Software (Version 9.55).

#### The surface area of the AC

The surface area was analyzed by Brunauer, Emmett, and Teller's (BET) method. About 4 g activated carbon was placed in three degassed sample tubes at $100 ^\circ \mathrm{C}$ for 2 h. The adsorption and desorption of N_2_ gas at 700 mm atmospheric pressure determines the surface area using a surface area analyzer (SA-9600 Horiba, Japan) [22, 58].

### Determination of pH at point of zero charge (pH_PZC_)

The point of zero charges of the AC was determined using the salt addition method. A 50 mL 0.01 M NaCl solutions was prepared in nine 250 mL Erlenmeyer flasks. Their initial pH is then adjusted in the range of 2 to 10. The pH was adjusted using 0.01 M HCl and 0.01 M NaOH solution. A 0.5 g of AC was then added to each solution and mixed at room temperature ($25 ^\circ \mathrm{C}$) using an orbital shaker at 150 rpm for 36 h. Then, the final pH of the solutions was recorded and the change in pH (pH_f_-pH_i_) was calculated. Finally, a graph of change in pH versus initial pH was plotted to determine the pH_PZC_ at the x-intercept [59].

### Optimization of Cr (VI) adsorption

The experiments were conducted in batch mode at room temperature ($25 ^\circ \mathrm{C}$ ± 1). Four experimental factors with three levels were selected, as shown in Table 1. The pH of the solution, adsorbent dose, initial Cr (VI) concentration, and contact time was set as factors. These factors were based on a previous study, which examined the removal of Cr (VI) using microporous activated carbon [60]. Removal efficiency was set as a response variable. Based on factorial experimental design, four factors with three levels will give (3^4^) 81 runs. However, the runs were fixed to 30 by Design Expert ®software version 12.0 using central composite design (CCD) under the response surface methodology (RSM) approach. The total number of experiments was calculated using the following equation:

5 $$ {\text{N}} = { }2^{{\text{F}}} + { }2{\text{F }} + { }x_{0} $$

where N is the number of experimental runs, F represents the factor number, and xo is the number of replicates at the central point.

**Table 1:** Adsorption factors with their levels under full factorial design

Factors	Levels		
	Low (−)	Middle (0)	High ( +)
pH	4	5	6
Adsorbent dose (g)	1	2	3
Initial concentration (mg/L)	0.5	2.25	4
Contact time (min)	60	90	120

In this study, N is 30, F is 4 and x_o_ is 6. All batch experiments were carried out in 250 mL Erlenmeyer flasks with 200 mL solution (adsorbate + adsorbent) in an orbital shaker at 300 rpm/min. The solution was filtered using grade 42 Whatman filter paper and the concentration of Cr (VI) ions was determined using UV-spectrophotometer (JASCO V-770) at the wavelength of 540 nm using diphenylcarbazide (DPC) as the complexion agent.

The removal efficiency and adsorption capacity (mg/g) was calculated using the following formula.

6 $$ {\text{Re \% }} = \left( {\frac{co - ce}{{co}}} \right) \times 100 $$

7 $$ {\text{qe}} = \frac{co - ce}{m} \times v $$

where Re is the removal efficiency, q_e_ is the adsorption capacity, *C*o is the initial Cr (VI) concentration (mg/L), Ce is the final Cr (VI) concentration (mg/L), V is the adsorbate volume (L), and m is the mass of adsorbent (g).

The quadratic regression model established by response surface methodology under CCD was used to conduct the regression analysis. The quadratic model is given by the following formula:

8 $$ {\text{R}} = \,{ }\beta {\text{o }} + { }\sum\nolimits_{{\text{i = 1}}}^{{\text{k}}} {\beta {\text{ixi}}\,} + \,\sum\nolimits_{{\text{i = 1}}}^{{\text{k}}} {\beta {\text{ii}}\left( {{\text{xi}}} \right)}^{2} \, + \,\sum\nolimits_{{\text{i = 1}}}^{{{\text{k}} - 1}} {\sum\nolimits_{{\text{j = i + 1}}}^{{\text{k}}} {\beta {\text{ijxixj}}} } $$

where R is the predicted response, β0 the constant coefficient, β*i* the linear effect coefficients, β*ii* the quadratic effect coefficients, β*i*j the interaction effect coefficients, *xi*, and *x*j are the independent variables and K is the number of independent variables.

### Effect of individual factors

The effect of pH, adsorbent dose, initial Cr (VI) concentration, and contact time on Cr (VI) removal was studied by varying one factor while keeping the others constant at optimum points as shown in Table 2. The experiment was carried out in a 200 mL solution using a 250 mL Erlenmeyer flask at room temperature and at 300 rpm/min. The concentration of Cr (VI) in the filtrate was determined by UV-spectrophotometer using DPC as the complexion agent.

**Table 2:** Individual factor values for the adsorption experiment

Factor	pH	Adsorbent dose (g)	Contact time (min)	Initial Cr (VI) concentration
1	4, 4.5, 5, 5.5, 6	2	90	2.25
2	5	1, 1.5, 2, 2.5, 3	90	2.25
3	5	2	60, 75, 90, 105, 120	2.25
4	5	2	90	0.5, 1.5, 2.25, 3.5, 4

### Adsorption isotherm

Adsorption isotherms were studied by taking 200 mL of Cr (VI) solutions at different initial.

concentrations (0.5, 2.25, 3.5, and 4 mg/L) in 250 mL Erlenmeyer flasks. The optimum pH was adjusted for each solution. Then, the optimum adsorbent dose was added to the solutions and agitated at 300 rpm/min for the optimum duration in an orbital shaker at room temperature. By using DPC as a complexion agent, the filtrate was analyzed using UV-spectrophotometer. To establish the adsorptions capacity of the adsorbent, experimental data were fitted against isotherm equations of the Langmuir and Freundlich models. Langmuir adsorption isotherm model assumes that the adsorption energy is the same at the entire site and that the adsorption molecules cannot be migrated across the surface or interact with the neighboring molecules. The linear and nonlinear equation of Langumer isotherm model is presented in Eq. 9 and 10 respectively. Moreover, the separation constant (*R*L) was measured using Eq. 11.

9 $$ \frac{{{\text{C}}_{{\text{e}}} }}{{{\text{q}}_{{\text{e}}} }} = \frac{1}{{{\text{q}}_{{\text{m}}} KL}} + \frac{{{\text{C}}_{{\text{e}}} }}{{{\text{q}}_{{\text{m}}} }} $$

10 $$ qe = \frac{qm*KL*Ce}{{1 + KL*Ce}} $$

where q_m_ (mg/g) is the maximum adsorption capacity of (Cr VI) per unit mass of activated carbon, K_L_ (L/g) is the Langmuir constant, which is related to the equilibrium adsorption constant, Ce (mg/L) and qe (mg/g) are the equilibrium values of Cr (VI) concentration and adsorption capacity, respectively.

11 $$ {\text{RL}} = \frac{1}{1 + KLCo} $$

where *C*o (mg/L) is the initial Cr (VI) concentration.

The Freundlich isotherm equation expressed adsorption on a heterogeneous surface. The linear model equation in terms of logarithm function is presented in Eq. 12 and the nonlinear equation is presented in Eq. 13.

12 $$ {\text{logq}}_{{\text{e}}} = {\text{logK}}_{{\text{f}}} + { }\frac{1}{{\text{n}}}{\text{logC}}_{{\text{e}}} $$

13 $$ qe = KfCe^{\frac{1}{n}} $$

where qe (mg/g) is the equilibrium loading in, Ce is the equilibrium concentration in mg/L, K_f_ (L/g) is the Freundlich constant, and n is the adsorption intensity.

### Adsorption kinetics and intraparticle diffusion

Adsorption kinetics express the solute removal rate that controls the residence time of the adsorbate in the solid-solution interface. The adsorption rate is mainly determined by the rate of molecules' arrival at the adsorbent surface and by the proportion of adsorbed molecules. About 200 mL of solution was prepared in a 250 mL Erlenmeyer flask. The pH, adsorbent dose, and initial Cr (VI) concentration were adjusted at optimum points. The solution was agitated at 300 rpm in an orbital shaker at room temperature. The solution was sampled at the time interval of 60, 75, 90, and 120 min, and the filtrate was analyzed using UV-spectrophotometer and DPC as a complexing agent. The experimental data were analyzed against the kinetics models. These models explain the mechanism of the adsorption process. The pseudo-first-order and pseudo-second-order model equations are expressed as follows:

14 $$ {\text{log}}\left( {{\text{qe}} - {\text{qt}}} \right) = logqe - \frac{k1t}{{2.303}} $$

15 $$ \left( \frac{t}{qt} \right) = \frac{1}{{k2*\left( {qe} \right)^{2} }} + \left( \frac{t}{qe} \right) $$

16 $$ \left( {{\text{qt}}} \right) = { }\left( {\frac{{{\text{Co}} - {\text{Ct}}}}{{\text{m}}}} \right){\text{*V}} $$

where qt and qe (both in mg/g adsorbent) are the amounts of Cr (VI) adsorbed at time t and equilibrium, respectively; K_1_ (g/mg min) is the pseudo-first-order sorption rate constant; K_2_ (g/mg min) is the pseudo-first-order sorption rate constant, V is the volume of the solution (L), m is the mass of adsorbent (g) and t is contact time (min).

The diffusion mechanism of Cr (VI) was studied using Weber–Morris intraparticle diffusion model as shown in Eq. 15. This model expresses whether intraparticle diffusion is the only rate control step in the diffusion of Cr (VI) or not. Moreover, it shows whether the intragranular or extragranular diffusion process is the main control adsorption process [61].

17 $$ qt = kid*t^{0.5} + c $$

where k_id_ (mg. g^−1^min^0.5^) is the intraparticle diffusion rate constant and* c* is a constant number.

**Fig. 2:**
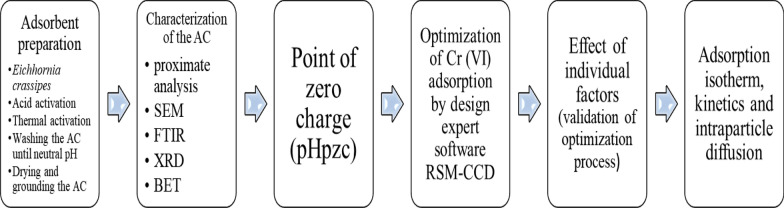
General methodology of the study

The process of the whole methodology is shown in Fig. 2.

## Results and discussion

### Characteristics of the adsorbent

The proximate analysis of the activated carbon in percentage units was moisture content (5.6%), volatile matter (18.2%), ash content (14.4%), and fixed carbon (61.8%), as shown in Table 3. The two most important parameters used to describe the quality of activated carbon are fixed carbon and ash [7]. An ideal activated carbon has a high percentage of fixed carbon, a small percentage of ash content, and medium content of volatile matter [5].

**Table 3:** Proximate analysis of the activated carbon

Parameters	Value (%)
Moisture	5.6
Volatile matter	18.2
Ash	14.4
Fixed carbon	61.8

The moisture content recorded in this study was higher than the value reported by Tessema et al*.* [5] while studying activated carbon prepared from *Canna indica*. It was also reported a higher moisture content than the value obtained in this study while studying activated carbon prepared from *Arundinaria Alpina* Stem. A higher value of volatile matter was recorded in this study than the value reported by Bedada et al*.* [55] while studying *Parthenium hysterophorus* weed [7]. A lower value of ash content was reported [62, 63] than the value obtained in this study while studying activated carbon prepared from *Typha orientalis* and grape stalk, respectively. A higher value of fixed carbon content was recorded in this study than the values reported [64]. However, a fixed carbon value of 68.73%, which is higher than the value obtained in this study [65].

**Fig. 3:**
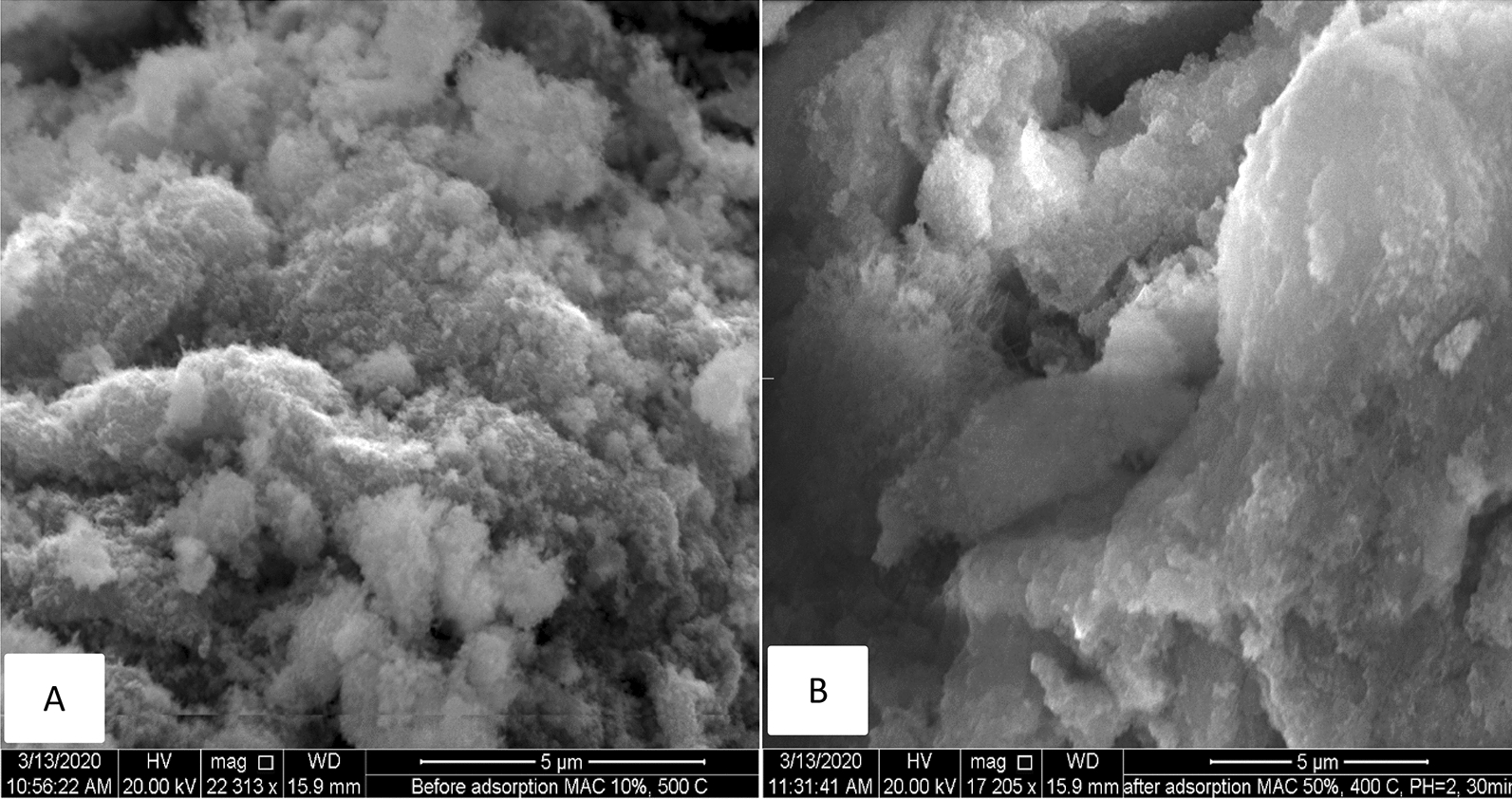
SEM analysis of activated carbon before adsorption (**A**) and after adsorption (**B**)

The SEM image before and after the adsorption of Cr (VI) from an aqueous solution was analyzed, as shown in Fig. 3. A highly porous structure with heterogeneous and irregular size and shape was observed in the micrograph. This allows the different sizes of the adsorbate to be adsorbed efficiently and effectively. The surface magnification for both before and after activation was 50 μm. As shown in Fig. 3A, the pour space created might be due to the chemical activation by H_2_SO_4_ and thermal activation at $500 ^\circ \mathrm{C}$. During carbonization, the evaporation of the chemical reagent (H_2_SO_4_) results from the increase in the number of pores. As shown in Fig. 3(B), the created pores become filled with Cr (VI) ions, which results from the reduction of the number of empty pores.

**Fig. 4:**
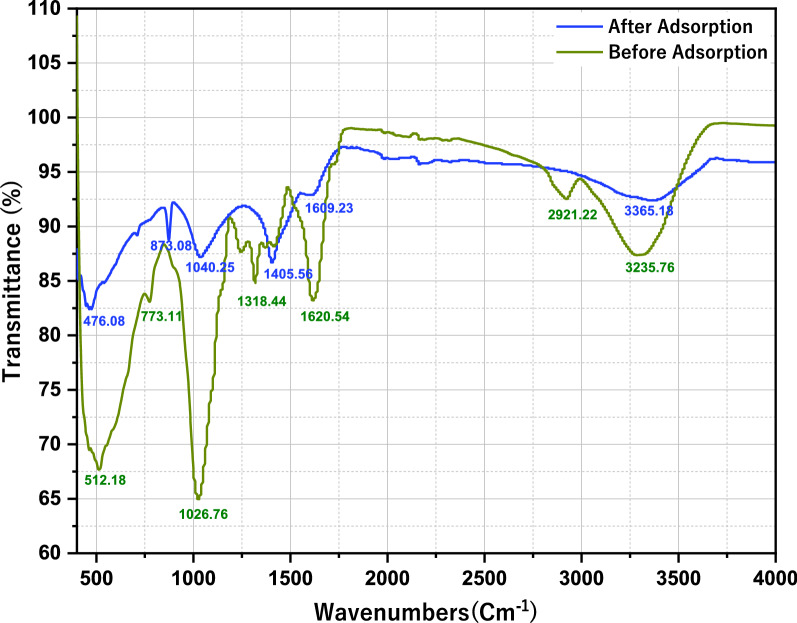
FTIR analysis of activated carbon before and after adsorption treatment

FTIR spectra were used to identify the surface functional groups of *Eichhornia crassipes* before and after adsorption. As shown in Fig. 4, seven clear peaks were observed before adsorption and six clear peaks were observed after adsorption. The presence of many peaks indicates the complex nature of the adsorbent with functional groups.

FTIR analysis before adsorption indicates that the peak obtained at 3235.76 cm^−1^ is assigned to the (O–H) hydroxyl group [66]. The peak obtained at 2921.22 cm^−1^ originated from asymmetric (C–H) stretching of the methyl group. The peak value at 1620.54 cm^−1^ corresponds to (C = C) stretching of the alkene and aromatic groups. The peck obtained at 1318.44 cm^−1^ was attributed to the (C–O) stretching of the phenolic group [67]. The peak value obtained at 1026.76 cm^−1^, 773.11 cm^−1^, and 512.18 cm^−1^ indicates the presence of Alkyl ketone, (C–Cl) chloride, and (R-X) alkyl halides, respectively. The shift of band wavenumbers (cm^−1^) before and after adsorption is presented in Table 4. The different peaks observed before and after adsorption might be due to the presence of various functional groups.

**Table 4:** FTIR spectra characteristics of *Eichhornia crassipes* before and after adsorption

FTIR Peak	Assigned functional group	Band wavenumber (cm^−1^)		
		Before adsorption	After adsorption	Shift difference
1	(O–H) Hydroxyl	3235.76	3365.18	129.42
2	(C–H) Methyl	2921.22	0	2921.22
3	(C = C) Alkene and aromatic	1620.54	1609.23	11.31
4	(C–O) Phenolic	1318.44	1405.56	87.12
5	Alkyl ketone	1026.76	1040.25	13.49
6	(C–Cl) Chloride	773.11	873.08	99.97
7	(R-X) Alkyl halides	512.18	476.08	36.1

The binding of Cr (VI) ion in the active site of the activated carbon might be the reason for the differences in peak frequencies in the FTIR spectrum. The adsorption of Cr (VI) ion in the activated carbon derived from Eichhornia crassipes indicates that the activated carbon has the potential to interact with other cations.

**Fig. 5:**
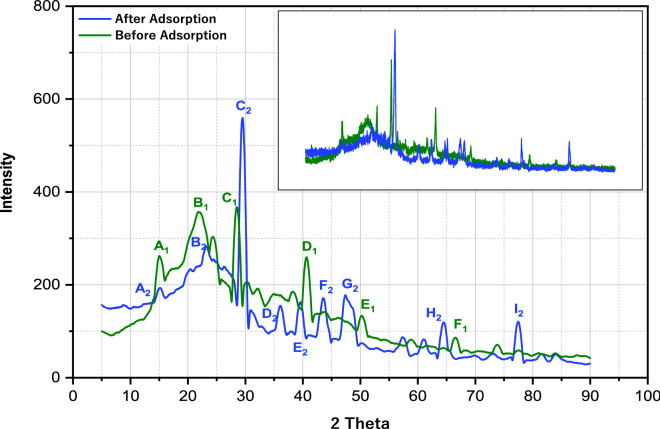
XRD analysis of activated carbon derived from *Eichhornia crassipes*

The presence of the amorphous and crystalline matrix before and after adsorption was assessed by X-ray diffraction, as shown in Fig. 5. Many peaks were observed in both adsorption analyses (before and after). Before adsorption, high-intensity peaks were observed at 2 theta of 15°, 23°, 28°, 41°, 50^o^, and 67°. After adsorption, significant peaks were observed at 2 theta at 15°, 24°, 29°, 36°, 40°, 43°, 47°, 64°, and 77°. In both adsorption analyses, the high-intensity peaks indicate the presence of crystalline structure [68]. As the 2 theta values increase in the spectra pattern, the intensity of peaks decreases, which results from the presence of an amorphous carbon arrangement [5].

The BET analysis shows that the surface area of *Eichhornia crassipes* before activation was 60.6 g/m^2^. After chemical and thermal activation of the material, the surface area increased to 794.2 g/m^2^. The increment in the number of pores might be due to the activation of the material by acid and heat [69, 70]. After the activated carbon adsorbs Cr (VI) ion, the surface area decreased to 412.6 g/m^2^. This might be due to the occupation of the pores by the Cr (VI) ion.

### Point of zero charge (pHpzc)

**Fig. 6:**
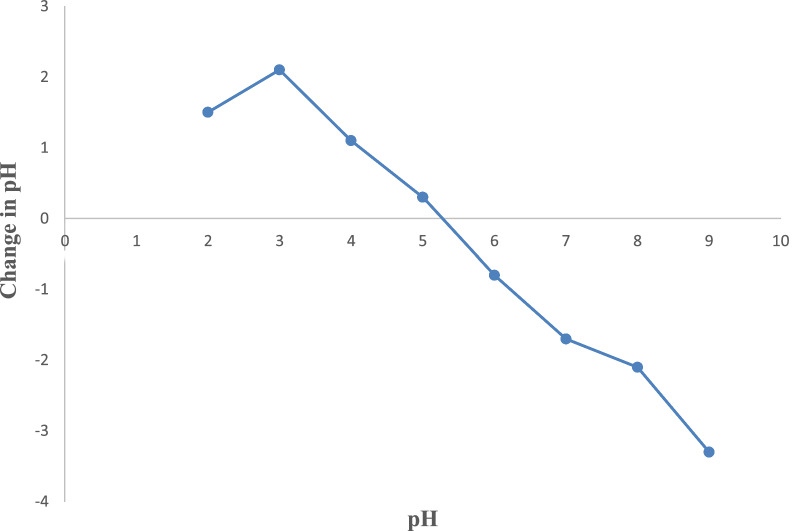
Point of zero charge (pHpzc)

When the pH is lower than the pzc value, the adsorbent surface is positively charged, which attracts anions (negatively charged ions). On the other hand, if the pH is higher than the pzc, the surface of the adsorbent is negatively charged, which indicates that the surface attracts cations (positively charged ions) [71]. The point of zero charge (pHpzc) on the surface of the AC is 5.20 as shown in Fig. 6. The Cr(VI) removal is favorable at pH value higher than pHpzc. The anion (HCrO_4_^−^) adsorption is favorable at pH value lower than pHpzc [59].

### Optimization of Cr (VI) removal

The actual and predicted values of Cr (VI) removal in percentages is presented in Table 5.

**Table 5:** The experimental and predicted value of Cr (VI)

Run	pH	Contact time (min)	Dose (g/200 mL)	Initial concentration (mg/L)	Cr (VI) Removal (%)	Predicted Cr (VI) Removal (%)
1	5	90	3	2.25	95.57	95.65
2	6	90	2	2.25	89.67	90.05
3	5	60	2	2.25	92.14	92.55
4	6	60	3	4	79.87	79.85
5	5	90	1	2.25	94.04	94.81
6	5	90	2	0.5	95.27	94.76
7	4	120	1	0.5	85.89	86.03
8	4	60	1	0.5	81.49	81.03
9	4	120	1	4	78.49	78.51
10	6	120	1	0.5	83.21	83.36
11	6	120	3	4	78.54	78.67
12	5	90	2	2.25	98.37	97.59
13	5	90	2	2.25	98.37	97.59
14	6	120	1	4	75.73	75.03
15	4	90	2	2.25	91.39	91.85
16	5	90	2	2.25	97.88	97.59
17	4	60	1	4	77.95	77.84
18	4	120	3	0.5	83.61	83.34
19	4	120	3	4	80.82	80.69
20	5	90	2	2.25	98.37	97.59
21	6	120	3	0.5	81.89	82.12
22	5	90	2	2.25	97.11	97.59
23	4	60	3	4	81.24	80.76
24	6	60	1	0.5	79.21	79.46
25	5	90	2	2.25	97.97	97.59
26	6	60	3	0.5	79.33	78.97
27	4	60	3	0.5	78.25	79.08
28	5	90	2	4	90.08	91.44
29	5	120	2	2.25	94.03	94.46
30	6	60	1	4	75.53	75.47

The maximum removal efficiency (98.37%) was achieved at pH 5, contact time of 90 min, adsorbent dose of 2 g, and initial Cr (VI) concentration of 2.25 mg/L. The minimum removal efficiency (75.5%) was found at pH 6, contact time of 60 min, solution volume of 200 mL adsorbent dose of 1 g, and initial Cr (VI) concentration of 4 mg/L.

### ANOVA and development of the regression model equation

The model adequacy was tested by determining the significant variables using analysis of variance (ANOVA) as shown in Table 6.

**Table 6:** ANOVA for response surface quadratic model for Cr (VI) removal

Source	Sum of Squares	df	Mean Square	F-value	p-value	
Model	1910.42	14	136.46	265.07	< 0.0001	
A-pH	14.49	1	14.49	28.15	< 0.0001	
B-Init. conc	49.67	1	49.67	96.48	< 0.0001	
C-Dose	3.19	1	3.19	6.20	0.0250	
D-Cont. time	16.44	1	16.44	31.93	< 0.0001	
AB	0.6521	1	0.6521	1.27	0.2781	
AC	2.14	1	2.14	4.15	0.0596	
AD	1.24	1	1.24	2.40	0.1419	
BC	23.74	1	23.74	46.12	< 0.0001	
BD	18.77	1	18.77	36.46	< 0.0001	
CD	0.5513	1	0.5513	1.07	0.3171	
A^2^	114.16	1	114.16	221.76	< 0.0001	
B^2^	52.30	1	52.30	101.60	< 0.0001	
C^2^	14.47	1	14.47	28.10	< 0.0001	
D^2^	43.19	1	43.19	83.90	< 0.0001	
Residual	7.72	15	0.5148			
Lack of fit	6.50	10	0.6505	2.67	0.1448	not significant
Pure error	1.22	5	0.2435			
Cor total	1918.14	29				

Regression coefficients were used to examine the fitness of linear, quadratic, and cubic models (R^2^). A quadratic regression model with R^2^ = 0.98 was the best fit for the experimental data. Thus, the value of Cr (VI) removal was predicted using a quadratic model. The final equation, which is used to make predictions about the response for a given level of each factor, is described as follows:

18 $$ YCr\left( {VI} \right) = \,97.59 - 0.8922A - 1.66B + 0.4211C + 0.9556D + 1.22BC - 1.08BD - 6.64A^{2} - 4.49B^{2} - 2.36C^{2} - 4.08D^{2} $$

where Y_**Cr (VI)**_ is the Cr (VI) removal, A the pH, B is the initial concentration, C is the adsorbent dose, D is the contact time, BC, BD, A^2^, B^2^, C^2^, and D^2^ refer to the interaction effects of initial concentration and adsorbent dose, initial concentration and contact time, among pH, among initial concentration, among dose and among contact time, respectively.

The P and F values were used to determine the significance of the model. The model F-value of 265.07 indicates that the model is statistically significant. An F-value of this magnitude has a 0.01 percent chance of occurring due to noise. Model terms with P-values less than 0.0500 are significant [72]. A, B, C, D, BC, BD, A^2^, B^2^, C^2^, and D^2^ are significant model terms in this situation. The model interaction values for AB, AC, AD, and CD, however, are insignificant (P > 0.05). As a result, to improve the model, the insignificant model interaction values were reduced from the model equation. Therefore, from the above equation, it can be noted that pH, initial Cr (VI) concentration, adsorbent dose, contact time, the interaction effect of initial concentration and adsorbent dose, and the interaction effect of initial concentration and contact time are the factors that significantly affect Cr (VI) removal from aqueous solution.

The F-value of 2.67 for the lack-of-fit indicates that it is not significant in comparison to the pure error. A lack-of-fit value that could arise owing to noise has a 14.48 percent chance of occurring. A non-significant lack of fit means that the model fits perfectly. The difference between predicted R^2^ (0.9828) and adjusted R^2^ (0.9922) was less than 0.2. This indicates that the predicted R^2^ is in reasonable agreement with the adjusted R^2^.

### Effect of individual factors

#### Effect of pH

**Fig. 7:**
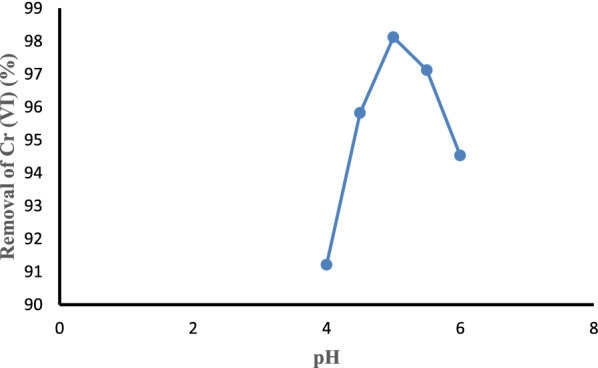
Effect of pH on Cr (VI) removal

pH is one of the factors that had a substantial effect on the adsorption of Cr (VI) ions. The effect of pH on the adsorption of Cr (VI) was studied by varying the pH from 4 to 6 while keeping the initial Cr (VI) concentration, adsorbent dose, and contact time constant at 2.25 mg/L, 2 g, and 90 min, respectively. The effect of pH can be evidenced by the drastic changes in adsorption percentage with different pH values. The pH affected the adsorption process through the dissociation of functional groups on the active sites of the surface of the adsorbent. At pH 4, the Cr (VI) ions removal was 91.21%. The adsorption increased to 98.12% because of the increase in pH from 4 to 5 as shown in Fig. 7. As pH increases, the number of anions increases on the surface, which provides a large number of negative sites for the adsorbate. The Cr (VI) removal efficiency decreases while increasing the pH from 5 to 6. At pH 6, the Cr (VI) removal becomes 94.53%. When pH increases, the repulsion force between negatively charged adsorbent and chromium anions (HCrO_4_^−^, Cr_2_O_7_^2−^, and CrO_4_^2−^) increases, which affects the removal efficiency [73].

#### Effect of adsorbent dose

**Fig. 8:**
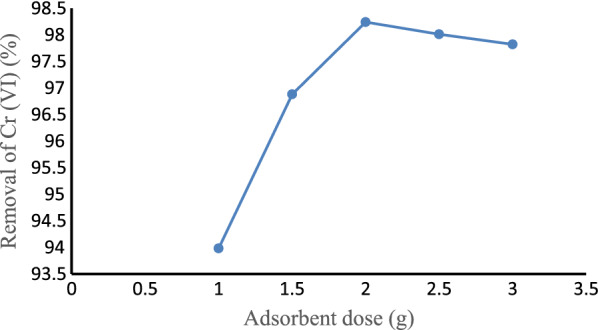
Effect of adsorbent dose on Cr (VI) removal

The Cr (VI) removal efficiency was studied by varying the adsorbent dose from 1 to 3 g while keeping the pH, initial Cr (VI) concentration, and contact time constant at 5, 2.25 mg/L, and 90 min, respectively as shown in Fig. 8. Adsorption of Cr (VI) increased from 93.98% to 98.24% when increasing the adsorbent dose from 1 to 2 g, respectively. This might be due to the increase in the availability of adsorption sites [74]. Further increase of the adsorbent dose to 3 g slightly decreases the efficiency to 97.82%. This might be due to the better occupation of lower energy sites in large fractions than the available higher energy sites [75].

#### Effect of contact time

**Fig. 9:**
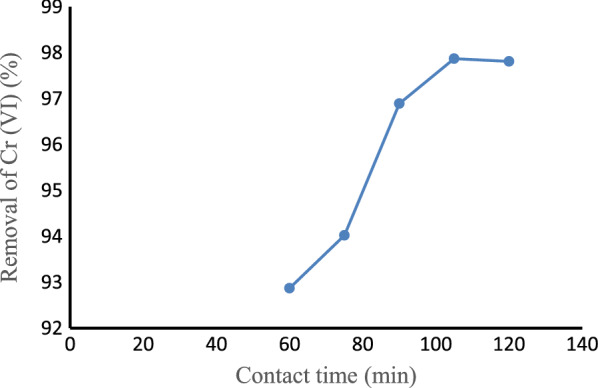
Effect of contact time on Cr (VI) removal

The effect of contact time on the removal of Cr (VI) was studied by varying the contact time from 60 to 120 min while keeping pH, adsorbent dose, and initial Cr (VI) concentration constant at 5, 2 g, and 2.25 mg/L respectively. As shown in Fig. 9, as the contact time increased from 60 to 105 min, the removal of Cr (VI) increased from 92.87% to 97.87%. This might be due to the fast adsorption of Cr (VI) ions on the external surface of the activated carbon at the initial stages [76]. The removal of Cr (VI) slightly decreased from 97.87% to 97.81% when the contact time increased from 105 to 120 min. The adsorption rate increased rapidly in the initial stages but slowed down later because of the attainment of equilibrium [77]. The equilibrium time for the adsorptions of Cr (VI) on activated carbon was found to be around 105 min.

#### Effect of initial Cr (VI) concentration

**Fig. 10:**
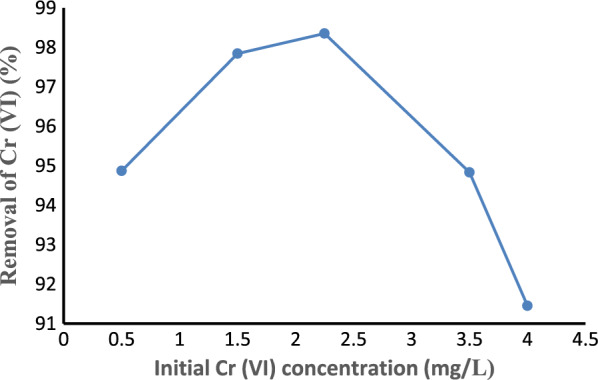
Effect of initial concentration on Cr (VI) removal

The effect of initial Cr (VI) concentration on Cr (VI) removal was studied by varying the initial concentration of Cr (VI) from 0.5 to 4 mg/L while keeping pH at 5, the adsorbent dose at 2 g, and the contact time constant at 90 min as shown in Fig. 10. The initial metal ion concentration offers a vital driving force to pass over all mass transfer resistances of the Cr (VI) ion between the aqueous solution and solid phases. As the initial concentration of Cr (VI) increased from 0.5 to 2.25 mg/L in the solution, the percentage of Cr (VI) removal efficiency increased from 94.87% to 98.35%. This might be due to the adsorption of Cr (VI) on the available surface area. This process continued up to the saturation point. The removal efficiency of Cr (VI) decreased to 91.45% as the initial Cr (VI) concentration increased from 2.25 to 4 mg/L. Since the number of available adsorption sites in the activated carbon is the same for all initial Cr(VI) ion concentrations, all adsorption sites will not be available anymore after reaching the saturation point, which leads to an increasing number of Cr (VI) ions that are not adsorbed [19].

### The interaction effects of factors on Cr (VI) removal

Out of the six interactions, only two were statistically significant, namely BC (the interaction effect of initial Cr (VI) concentration and adsorbent dose) and BD (the interaction effect of initial Cr (VI) concentration and contact time).

#### Initial Cr (VI) concentration and adsorbent dose

**Fig. 11:**
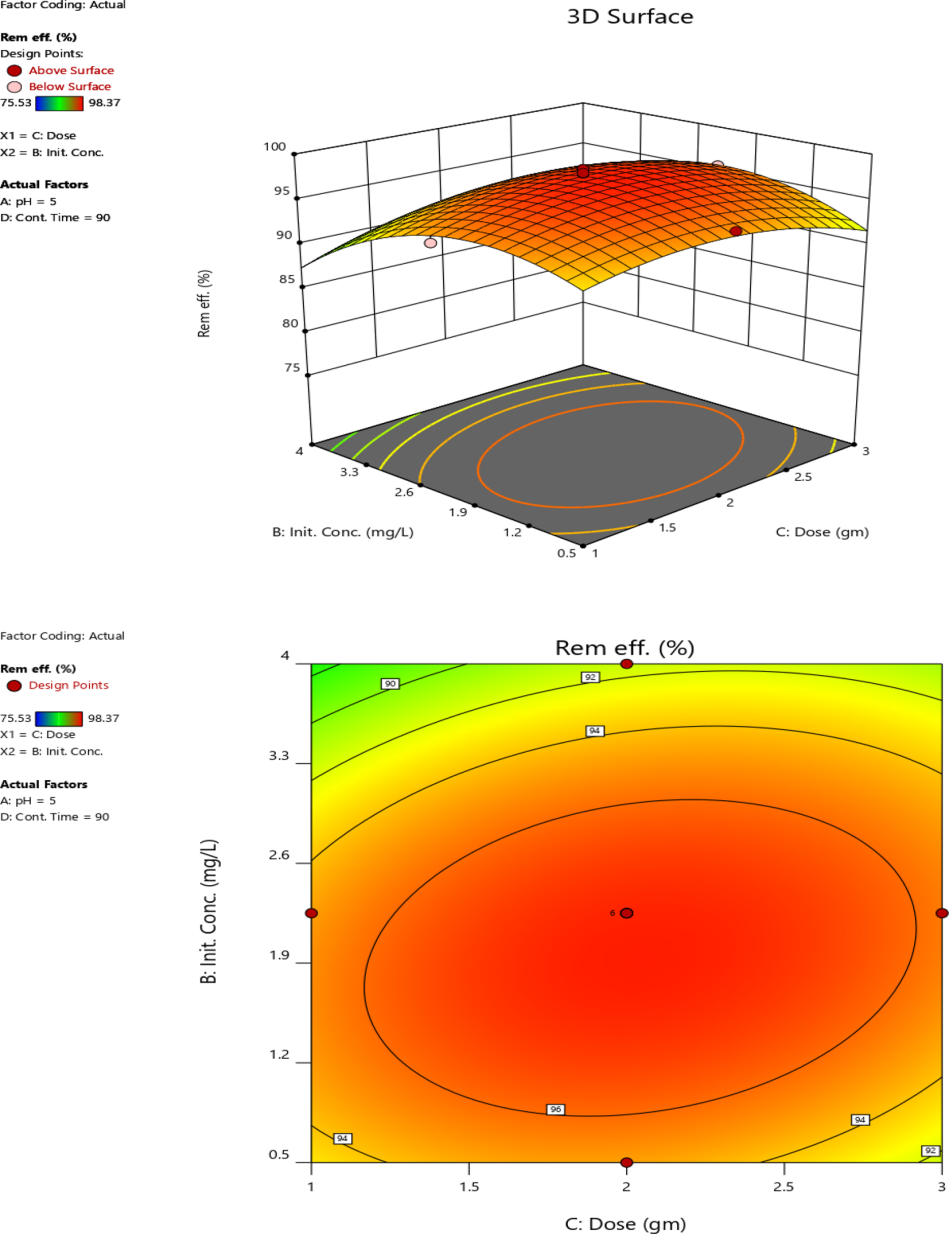
The interaction effect of adsorbent dose and initial concentration

The interaction effect of adsorbent dose and initial Cr (VI) concentration on Cr (VI) removal at constant pH (5) and contact time (90 min) is presented in the form of contour and three-dimensional response surface plots as shown in Fig. 11. As the initial concentration and adsorbent dose increased, the Cr (VI) removal efficiency increased up to some point**.** However, it declined when the initial concentration and the adsorbent dose increased further. The lowest Cr (VI) removal efficiency (90.08%) was observed at an adsorbent dose (2 g) and initial Cr (VI) concentration (4 mg/L). This might be due to the minimum availability of the adsorbent surface for Cr (VI) adsorption [78, 79]. Moreover, the available surface area might not be enough for the relatively higher concentration of Cr (VI) ions [80, 81]. The maximum Cr (VI) removal efficiency was obtained at a moderately medium adsorbent dose (2 g) and initial concentration (2.25 mg/L). The maximum Cr (VI) removal of the experiment was 98.37% and the predicted removal efficiency was 97.59%, which indicates the predicted and experimental removal efficiencies were in good agreement.

#### Initial Cr (VI) concentration and contact time

**Fig. 12:**
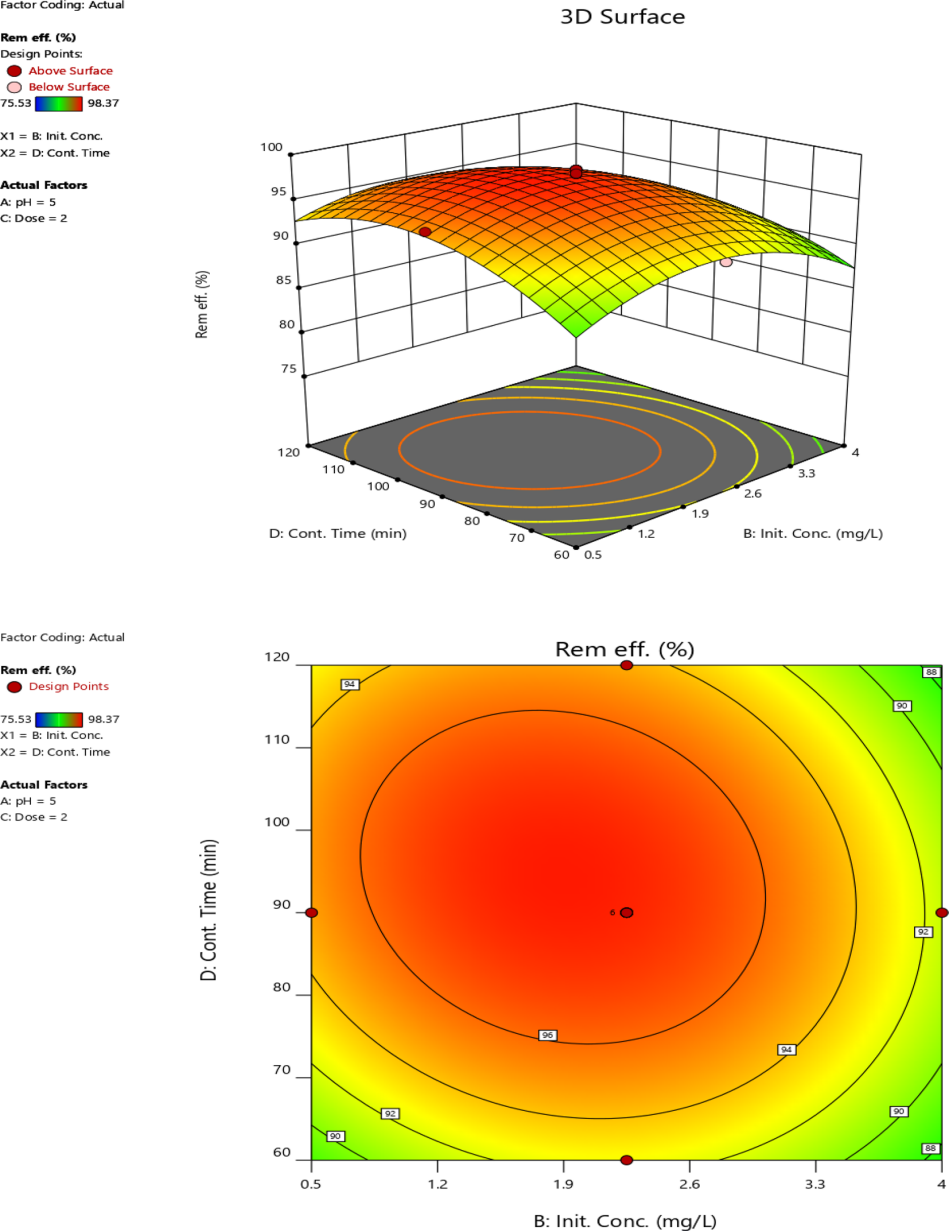
The interaction effect of initial concentration and contact time

The interactive effect of initial concentration and contact time on Cr (VI) removal efficiency at fixed pH (5) and adsorbent dose (2 g) is presented in the form of three-dimensional and contour plots as shown in Fig. 12. The Cr (VI) removal efficiency increased up to some point, but it declined when the initial concentration and contact time increased further. This might be due to the saturation of adsorption sites. Hence, with an increase in initial Cr (VI) concentration, no further adsorption could be achieved [77]. The minimum Cr (VI) removal efficiency (90.08%) was observed at the initial Cr (VI) concentration (4 mg/L) and contact time (90 min). The maximum Cr (VI) removal efficiency was found at medium values of initial Cr (VI) concentration (2.25 mg/l) and contacting time (90 min). The maximum Cr (VI) removal of the experimental site was 98.37% and the predicted removal efficiency at this point was 97.59%, indicating good agreement between the experimental and predicted removal efficiencies.

### Adsorption isotherm

To understand the adsorption mechanism, studying the adsorption isotherm of metal ions is very important. The adsorption isotherm describes the distribution of adsorbed molecules on the adsorbent interface. The linear and nonlinear regression method was used to determine the adsorption constants of the Langmuir and Freundlich models. In this study, the Langmuir isotherm model showed a relatively good correlation (R^2^ = 0.96 & 0.99) as shown in Table 7. This reveals that the adsorption process occurs in a single layer and at specific homogeneous adsorption sites on the adsorbent surface [82].

**Table 7:** Adsorption isotherm

Isotherm model		Linear	Non-linear
Langmuir	R^2^	0.9651	0.9988
	qm (mg/g)	0.6190	0.5128
	K_l_ (L/mg)	1	2.57
	Equation	Y = 1.6153X + 0.8251	$y=\frac{1.32X}{1+2.57X}$
Freundlich	R^2^	0.9438	0.9793
	n	1.1298	0.6098
	K_f_ ((mg/g) (L/mg)^1/n^)	2.4232	0.5625
	Equation	Y = 0.8851X + 0.0321	$y=0.5625{X}^{1.64}$

For different Co values (0.5, 2.25, 3.5, and 4 mg/L), RL values were 0.67, 0.51, 0.22, and 0.20, respectively. All RL values were between zero and one (0 < RL < 1). This indicates that the process is favorable [83].

### Adsorption kinetics and intraparticle diffusion

Pseudo-first-order and pseudo-second-order models were used to examine the kinetics of the metal adsorption process [84, 85]. As shown in Table 8, pseudo-second-order with an R^2^ value of 0.9956 best fits with the experimental data. This indicates that chemical reaction controls the adsorption kinetics. The intraparticle diffusion model indicates the diffusion mechanism. According to Yu et al*.* [86], in the qt vs t^0.5^ graph, if the regression line passes through the origin, the intraparticle diffusion process of Cr (VI) is the only rate control step. Moreover, if the regression line did not pass through the origin, the intragranular diffusion process of Cr (VI) is not the only rate control step. This indicates that other mechanisms also play a significant role in the rate control step. As shown in Table 8, the value of C is different from zero, which means the regression line did not pass through the origin. This indicates that extragranular diffusion processes such as surface adsorption and liquid film diffusion may affect the boundary layer control and adsorption rate [86].

**Table 8:** Adsorption kinetics and intraparticle diffusion

Kinetics model		
Pseudo-first-order	R^2^	0.5959
	K_1 (_min^−1^_)_	0.054
	Equation	Y = 0.0236X—3.2042
Pseudo-second-order	R^2^	0.9956
	K_2_ (g/mg/min)	0.8859
	Equation	Y = 25.294X + 723.47
Intraparticle diffusion	R^2^	0.9396
	C	0.0145
	K_ID_ (mg. g^−1^min^0.5^)	0.0400
	Equation	Y = 0.0016X + 13.017

This research is compared with other studies that are conducted on Cr (VI) removal in terms of removal efficiency as shown in Table 9.

**Table 9:** Comparison of this study with other studies

Precursor material	Removal efficiency (%)	References
Teff straw-based AC	95.90	[87]
Cassava sludge-based AC	98.22	[88]
Peyanum harmala seed-based AC	99.60	[89]
Powder AC modified by zero-valent iron and silver bimetallic nanoparticle	97.25	[90]
Corncob based AC	98.38	[91]
*Eichhornia crassipes*	98.40	This study

## Conclusions

Activated carbon derived from *Eichhornia crassipes* was used to remove Cr (VI) from the aqueous solution. The activated carbon was prepared by chemical activation using 0.1 M H_2_SO_4_ at 1:1 acid-to-sample ratio followed by thermal activation at $500^\circ$C for 2 h. The characteristics of the activated carbon indicate that it has a good adsorption potential. The maximum removal efficiency of 98.4% was achieved at pH 5, contact time of 90 min, adsorbent dose of 2 g, and initial Cr (VI) concentration of 2.25 mg/L. The adsorption mechanism was checked by Langmuir and Freundlich isotherm models. The experimental data were best fitted with Langmuir adsorption isotherm with an R^2^ value of 0.99, which indicates a single layer and homogeneous adsorption process. The R_L_ value lies between 0 and 1, which indicates that the process is favorable. The kinetics of the adsorption process was checked using pseudo-first-order and pseudo-second-order models, and the diffusion mechanism was determined by the intraparticle diffusion model. Pseudo-second order with an R^2^ value of 0.99 best fits with the experimental data. This indicates that chemical reaction controls the adsorption kinetics. In the intraparticle diffusion model, the value of C is different from zero, which indicates in addition to intraparticle diffusion, the extragranular diffusion process may also affect the boundary layer control and adsorption rate. Generally, it can be concluded that the activated carbon derived from *Eichhornia Crassipes* has a promising potential to remove Cr (VI) ions effectively and efficiently from wastewater. However, further investigation such as point of zero charge of the adsorbent, effect of mixing speed, thermodynamics study and column analysis have to be conducted before using it at the industrial level for wastewater treatment.

## Sample collection permission

Experimental research and field studies on plants, including the collection of plant material complied with the institution’s guidelines and regulations. During sample collection, our institute gave a support letter to give permission for sample collection and the textile industry owners gave permission for sample collection and in situ measurements. The voucher plant specimen was deposited in a university herbarium and the plant specimen was collected by the researchers and crossed checked against the herbarium.

## Data Availability

All data are fully available without restriction from the corresponding author at any time via email.
